# Optimized delivery of fluorescently labeled proteins in live bacteria using electroporation

**DOI:** 10.1007/s00418-014-1213-2

**Published:** 2014-04-03

**Authors:** Marko Sustarsic, Anne Plochowietz, Louise Aigrain, Yulia Yuzenkova, Nikolay Zenkin, Achillefs Kapanidis

**Affiliations:** 1 Clarendon Laboratory, Biological Physics Research Group, Department of Physics, University of Oxford, Parks Road, Oxford, OX1 3PU UK; 2 The Centre for Bacterial Cell Biology, Institute for Cell and Molecular Biosciences, Newcastle University, Baddiley-Clark Building, Richardson Road, Newcastle upon Tyne, NE2 4AX UK

**Keywords:** Electroporation, Single-molecule fluorescence, Live-cell imaging, Organic fluorophores

## Abstract

Studying the structure and dynamics of proteins in live cells is essential to understanding their physiological activities and mechanisms, and to validating in vitro characterization. Improvements in labeling and imaging technologies are starting to allow such in vivo studies; however, a number of technical challenges remain. Recently, we developed an electroporation-based protocol for internalization, which allows biomolecules labeled with organic fluorophores to be introduced at high efficiency into live *E. coli* (Crawford et al. in Biophys J 105 (11):2439–2450, 2013). Here, we address important challenges related to internalization of proteins, and optimize our method in terms of (1) electroporation buffer conditions; (2) removal of dye contaminants from stock protein samples; and (3) removal of non-internalized molecules from cell suspension after electroporation. We illustrate the usability of the optimized protocol by demonstrating high-efficiency internalization of a 10-kDa protein, the ω subunit of RNA polymerase. Provided that suggested control experiments are carried out, any fluorescently labeled protein of up to 60 kDa could be internalized using our method. Further, we probe the effect of electroporation voltage on internalization efficiency and cell viability and demonstrate that, whilst internalization increases with increased voltage, cell viability is compromised. However, due to the low number of damaged cells in our samples, the major fraction of loaded cells always corresponds to non-damaged cells. By taking care to include only viable cells into analysis, our method allows physiologically relevant studies to be performed, including in vivo measurements of protein diffusion, localization and intramolecular dynamics via single-molecule Förster resonance energy transfer.

## Introduction

Single-molecule fluorescence methods can be used to study a variety of cellular phenomena, such as interactions between specific molecules, their stoichiometry, dynamics and cell localization (Xie et al. 2008). Such studies are normally carried out using the green fluorescent protein and its variants (Tsien 1998). Fluorescent proteins allow simple and specific labelling of any protein of interest with high efficiency, and they are commercially available in various forms with different spectral, photophysical and chemical properties. However, fluorescent proteins are characterized by relatively poor photostability, which can present an issue for single-molecule localization against the high cellular autofluorescence background, and for single-molecule tracking on longer timescales (Dempsey et al. 2011; Shaner et al. 2005). Their large size can interfere with dynamics of the protein of interest, and intramolecular labeling may interfere with the structure of the protein or disrupt native interactions. Similarly, immune-fluorescence labeling requires the addition of at least one if not two antibodies, each of them larger than a single fluorescent protein (Sauer 2013). Until recently, the best alternative method available was intracellular labeling of proteins fused to polypeptide tags (e.g. SNAP, HALO, or TMP tags) but this still implies large protein fusions (e.g., SNAP tag: 20 kDa; Jones et al. 2011; Hinner and Johnsson 2010; Keppler et al. 2003; Wombacher et al. 2010). Due to their large size and labeling restrictions, fluorescent proteins and protein tags are also incompatible with measurement of intramolecular distances by single-molecule Förster resonance energy transfer (FRET). Organic fluorophores, which are typically used for in vitro applications, may in fact be better suited for many of the single-molecule applications in live cells. They are brighter and more photostable than fluorescent proteins (Dempsey et al. 2011; Shaner et al. 2005; Giepmans et al. 2006), ~100 times smaller in volume, and they can in principle be attached to any residue position in the protein, as long as the activity of the protein is preserved.

**Fig. 1:**
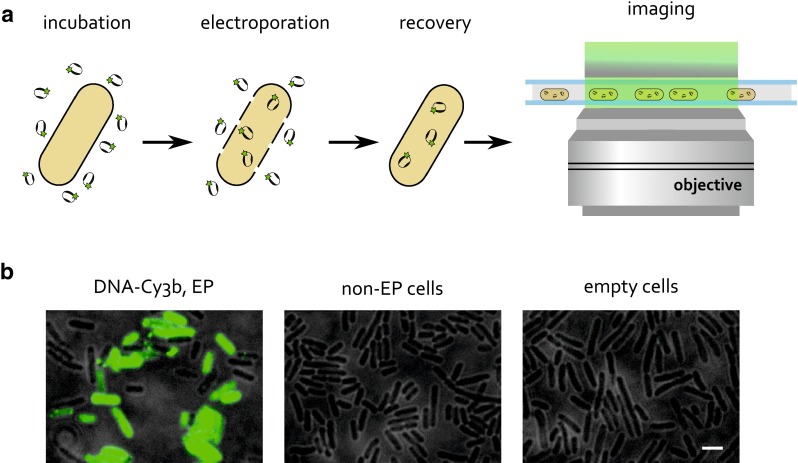
Internalization of fluorescently labeled molecules by electroporation. **a** Electrocompetent cells are incubated with the fluorescently labeled biomolecule, and electroporated with high-voltage electric field. Transient pores are formed in the cell membrane, allowing the molecule to be internalized. Cells are recovered in a rich medium, and thoroughly washed to remove non-internalized molecules. Imaging is performed on a fluorescence microscope set-up using either widefield or near-TIRF mode. **b** Example fields of view for internalization of 1 μM 45-bp DNA-Cy3b, at 1.4 kV voltage. Near-TIRF mode, 532-nm excitation at 600 μW, 100 ms exposure. Negative controls are also shown: ‘non-EP cells’ are cells that are incubated with DNA-Cy3b but not electroporated, and ‘empty cells’ are cells that are neither incubated with DNA-Cy3b nor electroporated. *Scale bar* 3 μm

However, since organic fluorophores are not compatible with genetic tagging, a means of internalization of organically labeled proteins into cells is required for in vivo studies. Internalization has been demonstrated in eukaryotic cells by means of scrape loading, syringe loading and microinjection (McNeil et al. 1984; Clarke and McNeil 1992; Taylor and Wang 1978; Sakon and Weninger 2010), but is not applicable to bacterial cells due to their small size and the presence of the cell wall. However, several methods that have classically been used for bacterial transformation, such as electroporation or heat shock (Dower et al. 1988; Neumann et al. 1982), appeared promising for internalization of fluorescent molecules. Heat-shocking cells has been employed to internalize short fluorescent DNAs (Fessl et al. 2012), but has not turned out to be effective for internalization of proteins. In our laboratory, we have recently developed a protocol for internalization by electroporation (Crawford et al. 2013; Fig. 1a). The method has been thoroughly characterized for delivery of short DNA fragments into *E. coli,* and has also demonstrated delivery of proteins of up to 100 kDa in size. Figure 1b shows typical data obtained for internalization of green-labeled DNA. High internalization efficiencies are achieved (up to 500 molecules per cell; Crawford et al. 2013), although there is a broad distribution of internalized molecules per cell. Non-electroporated cells, which are incubated with the fluorescent molecule but not electroporated, constitute an important negative control as they show no significant fluorescence, indicating successful washing-off of non-internalized molecules. Similarly, the background autofluorescence of cells, measured in cells that are neither incubated with the fluorescent molecule nor electroporated (‘empty cells’), is significantly below the fluorescence of electroporated cells.

Whilst our electroporation protocol has been used to deliver specific proteins into *E. coli*, we have noted that internalization of proteins faces several issues that deserve further method development. These include the use of high-salt buffers that are not compatible with electroporation conditions and may lead to arcing; the presence of contaminating (unreacted) dye in stock protein samples; and the presence of non-internalized fluorescent molecules that persist in the cell suspension even after thorough cell washing. In the following report, we address these issues and examine possible solutions, in order to provide general guidelines that can be followed when using electroporation to internalize any small or medium-sized fluorescently labeled protein into bacteria. Using our guidelines, we optimize the preparation and internalization of a small protein, the ω subunit of RNA polymerase. We also demonstrate the effect of electroporation voltage on the internalization efficiency of RNAP ω, as well as on viability of electroporated cells. This study should serve as a reference for choosing optimal conditions when using electroporation to internalize organically labeled proteins into live bacteria, depending on the application of interest and the desired internalization efficiency.

## Materials and methods

### Sample preparation

The double-stranded DNA oligonucleotide STD45T was prepared by automated synthesis (IBA GmbH). The sequence used was 5′**T**AAATCTAAAGTAACATAAGGTAACATAACGTAAGCTC-ATTCGCG-3′, where the underlined T base was labeled with Cy3b-NHS ester (GE Healthcare), as previously described (Crawford et al. 2013).

DNA Pol I and Klenow fragment (KF) were expressed, purified and fluorescently labeled as described (Joyce and Derbyshire 1995; Joyce et al. 2008; Santoso et al. 2010). Labelling efficiencies, quantified from UV–Vis spectra, were between 75 and 90 %. For electroporation, labeled proteins were dialysed into 50 mM Tris pH 7.4, 25 mM NaCl, 1 mM DTT, 50 % glycerol and stored at −20 °C.

C-terminal His_6_-tagged ω [Cys68] was expressed using pET expression system and purified by Ni^2+^-affinity chromatography in denaturing conditions. ω [Cys68] was reduced either as described in (Kim et al. 2008) or using Reduce-Imm (Pierce) column as per manufacturer’s instructions. Cy3b-labeled ω [Cys68] was prepared using Cy3b maleimide dye (Amersham) as in (Kim et al. 2008). Cy3-ω [Cys68] was purified from unincorporated dye by Ni^2+^-affinity chromatography.

### Analysis and removal of dye contamination

Fluorescent protein samples containing dye contaminants were added to 4–6 × recommended amount of Ni–NTA resin (10–15 mg/ml binding capacity), and incubated on a rotating wheel for 30 min at 4 °C. The resin was washed with 100 column volumes (CV) of 50 mM Tris pH 7.1, 25 mM NaCl, 10 mM imidazole, and the fluorescent protein eluted with 20 CV of 50 mM Tris pH 7.1, 25 mM NaCl, 200 mM imidazole. Eluate was dialysed into 50 mM Tris pH 7.5, 25 mM NaCl, 1 mM DTT, 50 % glycerol and stored at −20 °C. Fractions from different steps of the purification procedure were run on a denaturing SDS-PAGE gel (Mini-PROTEAN TGX Precast Gels; Bio-Rad), using a transparent sample buffer (250 mM Tris pH 6.8, 20 % glycerol, 2 % SDS, 1 mM DTT). In-gel fluorescence was imaged (Molecular Imager PharosFX Plus System; Bio-Rad), and the gels stained with Coomassie Brilliant Blue to confirm the identity of protein bands. Fluorescent bands were quantified in Fiji image-processing software using the ‘Plot Profile’ function, and the peaks integrated in OriginPro.

### Internalization by electroporation

Electrocompetent cells (Electro MAX DH5α-E; Invitrogen) were diluted 1:1 in water and stored in 20 μl aliquots. Fluorescently labeled DNA or protein was added to an aliquot of cell suspension at 50 nM–2.5 μM concentration and transferred to a pre-chilled 1-mm electroporation cuvette. For dye-contamination experiments, free Cy3b-maleimide dye was inactivated with 10 mM DTT for 10 min and diluted in water before being added to cells. Electroporation was performed at 1.0–1.8 kV in a standard electroporator (MicroPulser; Bio-Rad). Cells were recovered by incubation with 500 μl pre-warmed super optimal broth with catabolite repression (SOC) for 3 min at 37 °C. After recovery, cells were pelleted for 1 min at 3,300×*g* and 4 °C, and washed with phosphate buffered saline (PBS) solution containing 100 mM NaCl and 0.005 % Triton X100. Washing was repeated 2 more times with the same buffer, and 3 more times with PBS only. In the case of cell filtration, cells were transferred to an Ultrafree-MC centrifugal filter tube (0.22 μm pore diameter) after the first wash and spun 3× for 3 min at 800×*g* and 4 °C. In the case of internalization and viability analysis, cells were further recovered in EZ rich defined medium for 1–2 h at 37 °C. Non-electroporated control samples were treated identically except that no electroporation was performed. Empty-cell samples were prepared by diluting electrocompetent cells 5–10× in PBS. 5 μl of cells was applied to pads containing 1 % agarose (Bio-Rad Certified Molecular Biology Agarose) and 1× M9 minimal medium. In the case of internalization and viability analysis, M9 salts were replaced with EZ rich defined (fluorescence-friendly) medium to ensure cell growth and division.

### Buffer and protein-only electroporation

For buffer optimization experiments, buffers containing 50 mM Tris pH 7.4, 0–150 mM NaCl and 0–40 % glycerol were diluted 20× in water, to simulate the dilution under conditions of cell electroporation. Electroporation was performed at 1.0–1.8 kV in the absence of cells, using the same cuvette for each buffer condition, and the electroporation time constant was measured each time. Pure deionized water was tested for reference. For the aggregation assay, Pol I-Alexa647 sample was diluted in water to the same concentration as in cell electroporation experiments and electroporated under the same conditions (see above).

### Widefield and TIRF imaging

Samples were imaged on a customized inverted Olympus IX-71 microscope with a TIRF set-up. The pads were sandwiched between two coverslips and placed on the objective with the cell-covered side facing downwards. For internalization and viability analysis, the objective was heated to 37 °C (Objective Heater System; Bioptechs) to promote cell growth and division. Beams from a 532-nm Nd:YAG (Samba; Cobolt AB) and a 637-nm diode laser (Stradus; Vortran) were combined and collimated before focusing onto the back focal plane of the objective. The incident angle of the beam was adjusted such that either widefield or near-TIRF (also known as HILO; Tokunaga et al. 2008) illumination was achieved. Fluorescence from the sample was collected through the same objective, separated from the excitation light using a long-pass and a notch filter, and split into red and green channels using a dichroic mirror (630DRLP; Omega). The two channels were imaged onto separate halves of the chip of an electron-multiplying charge-coupled device (EM-CCD) camera (iXon +, 887-BI; Andor technology). Videos were recorded with manufacturer’s software, using the kinetic mode with 50–100 ms exposure. White light images were obtained using a white light lamp (IX2-ILL100; Olympus) and a condenser (IX2-LWUCD; Olympus) attached to the microscope as an illumination source.

### Internalization and viability analysis

Internalization images were obtained in Fiji by overlaying white-light (inverted) and fluorescence images (averaged over 10 frames, false coloured). Cells were segmented using an adapted version of programme ‘Schnitzcells’ (Young et al. 2012), and cell intensities quantified and normalized for the cell area by means of a custom-written MATLAB script. Intensities were corrected for the mean intensity of empty cells, to account for cellular autofluorescence. Viability was analysed by manually comparing white-light images of cells taken every 20–40 min, and classifying cells as growing/dividing, identical or damaged. For comparative analysis of loading and viability, the microscope stage was set (using Micromanager) to image an area of the pad first in the fluorescence mode for loading, and then in the white-light mode for viability analysis.

### Confocal microscopy

The electroporated sample was diluted to 100–200 pM in 40 mM Hepes–NaOH pH 7.3, 10 mM MgCl_2_, 1 mM DTT, 100 μg/ml BSA, 5 % glycerol, 1 mM mercaptoethylamine. Single-molecule measurements were performed at room temperature using a confocal microscope with alternating-laser excitation between a 532-nm and a 638-nm laser, as described (Doose et al. 2007; Selvin and Ha 2008). Two to four datasets of 10 min were recorded for each sample, photon streams were processed and burst search performed using custom-written software, as described (Santoso et al. 2010; Hohlbein et al. 2013).

## Results

### Buffer conditions for electroporation

Most proteins have a preference for specific buffer conditions, which ensure their structural stability and activity over time. At the same time, electroporation has to be carried out under conditions that ensure high protein internalization efficiency and maintain cell viability. In particular, the ionic strength of the buffer in which cells are electroporated should be high enough to maintain protein sample integrity but low enough to avoid the occurrence of an electrical short circuit (arcing). The electroporation time constant, which describes the exponential decay of the applied voltage with time, is indicative of whether a certain level of electrical discharge has occurred in the medium. For a standard electroporator (such as MicroPulser Electroporator; Bio-Rad), 4.00 ms can be taken as a conservative, and 3.00 ms as a liberal estimate for the lower bound of an acceptable electroporation constant that allows good cell loading and preserves cell viability. For reference, the electroporation constant of pure, deionized water is approximately 6.00 ms.

**Fig. 2:**
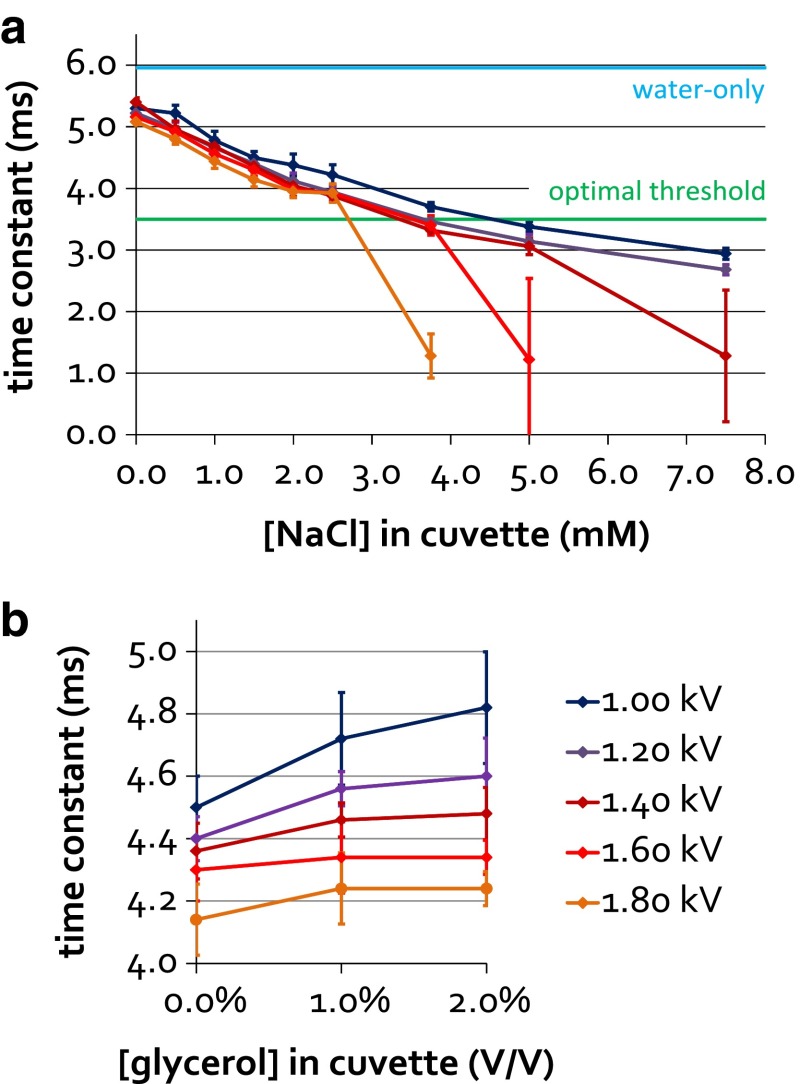
Effect of salt (**a**) and glycerol (**b**) concentrations in the stock buffer on electroporation time constant. **a** Buffer solutions containing 50 mM Tris pH 7.4 and 0–150 mM NaCl were diluted 20× in water, to simulate the dilution of stock protein solutions under conditions of cell electroporation. The NaCl concentrations shown refer to the final concentrations present in the electroporation cuvette, with 0–150 mM NaCl concentration range in the stock solution corresponding to 0–8 mM final concentration in the cuvette. The mean of 5 measurements at each buffer condition and each voltage is plotted, with the standard deviation represented by *error bars*. Occasional arcing events were excluded from data analysis except when they occurred consistently. The mean time constant for electroporation of water at 1.4 kV (6.00 ms) is shown with a *blue line*, and the optimal threshold for the time constant (3.50 ms) is indicated by a *green line*. **b** As in **a**, except that only 50 mM Tris pH 7.4, 30 mM NaCl buffer was used, with 0–40 % starting (0–2 % final) glycerol concentrations

To determine the highest salt concentration that can be used for protein electroporation, we measured the electroporation constant for buffers containing 50 mM Tris and between 0 and 150 mM NaCl, diluted 20 times in water. As anticipated, the time constant decreased with increasing salt concentration (Fig. 2a), and at higher concentrations there was a higher possibility of arcing occurring. Seeing that the electroporation voltage affects both the protein internalization efficiency and cell viability (discussed below), we also tested the effect of voltage on the electroporation time constant, and observed the constant to decrease with the increasing voltage. Whilst the effect was minor at low ionic strengths, it was significant at higher strengths. We conclude that a working buffer of 50 mM Tris (pH 7.4) and up to 50 mM NaCl (or equivalent) should be appropriate for successful electroporation at any voltage up to 1.80 kV. Higher ionic strength buffers may only be used with low voltage settings.

Finally, we noted that many of the protein storage buffers contain glycerol to maintain protein integrity and mitigate the effects of sample freezing and thawing. We therefore tested the effect of the concentration of glycerol in the buffer on the electroporation time constant. A minor but consistent positive effect was observed (Fig. 2b), suggesting that storing stock protein samples in a glycerol-based buffer is appropriate for the purposes of electroporation, and will not compromise cell loading.

### Dye contamination

Labeling of proteins with organic fluorophores involves incubation with a large excess of a reactive fluorophore, and although a dye-removal step (such as gel filtration) is normally included, some unreacted dye contamination often remains. This is either due to some of the free dye remaining in solution, or due to non-specific binding of the dye to the protein, either via hydrophobic or electrostatic interactions. Significant dye contamination is rarely observed for DNA fragments, as these have low affinity for non-specific binding of the dye, and because labeled DNAs are often purified from a polyacrylamide gel by cutting out a band of interest. Whilst dye contamination is not an issue for single-molecule in vitro experiments, it is a significant obstacle for electroporation-based internalization of proteins into cells. The reason lies in the small size of organic fluorophores compared to proteins, which causes fluorophores to be internalized at higher efficiency than proteins, and in the fact that the internalized free dye cannot be easily distinguished from labeled proteins inside the cell.

**Fig. 3:**
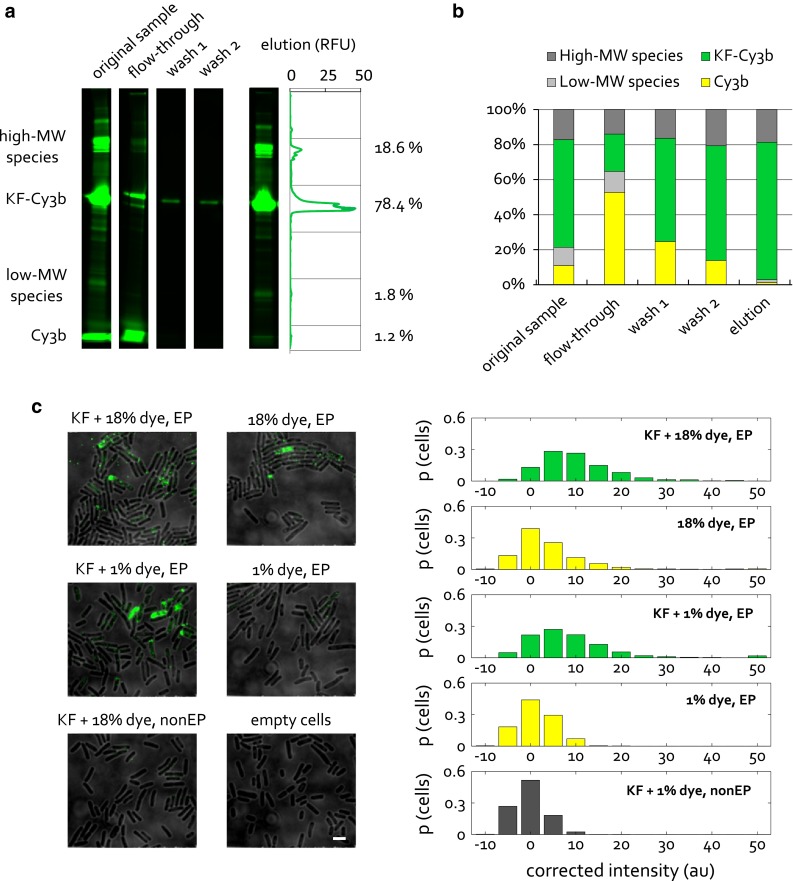
Analysis and removal of dye contamination from stock protein samples prior to electroporation. **a** In-gel fluorescence of an SDS-PAGE gel showing His-tag purification of KF-Cy3b. The different contaminants are marked: high-, low-MW protein species and free dye. Wash 1 corresponds to the fraction obtained after 30 CV, and wash 2 after 100 CV of buffer wash. Note that varying amounts of sample were loaded into the different wells of the gel, so band intensities should only be compared within the same lane. **b** The relative amounts of the different species present in the samples, quantified from the band intensities, as shown for the last lane in **a**. **c**, *left* Internalization of 1.5 μM KF-Cy3b before dye purification (18 % dye contamination) and after purification (1 % dye contamination), and internalization of Cy3b free dye at concentrations corresponding to 18 % and 1 % contamination. Empty cells and the non-electroporated control for KF-Cy3b before dye purification (18 % dye contamination) are also shown. Electroporation at 1.4 kV voltage, widefield mode, 637-nm excitation at 1 mW, 50 ms exposure. *Scale bar* 3 μm. **c**, *right* Distribution of cell-averaged (per-pixel) intensities, corrected for the mean fluorescence of empty cells, and given in proportion of the total cell count. >400 cells per sample were segmented

We devised a simple and quick protocol for the purification of dye contaminants from protein samples, which consists of binding the protein of interest to the Ni–NTA column, and washing the column with a large amount (100 CV) of buffer. Due to the large amount of buffer used, the washing is expected to be effective for removing both the free dye and the dye that is bound non-specifically to the protein. The latter may only be removed gradually, depending on the strength of the dye-protein interaction. We used KF, a 68-kDa fragment of *E. coli* DNA polymerase I, to develop our dye purification protocol. Figure 3a shows in-gel fluorescence for an SDS-PAGE gel of a typical His-tag purification of KF-Cy3b. The main protein species, the dye contaminant and other contaminants can all be distinguished, due to the high sensitivity of fluorescence imaging. The relative intensities of different bands in each lane can be extracted and correspond to the relative amounts of the different species in each sample (Fig. 3a, shown for the last lane). From this analysis, it is evident that His-tag purification of our fluorescently labeled protein results in a gradual but significant decrease in the amount of contaminating dye present (Fig. 3b). The lowest dye contamination that we could achieve was 1.2 %, ~15-fold lower than the starting amount of contamination. Notably, although high-molecular weight contaminants remain in our samples, they are unlikely to be an issue for internalization by electroporation as they will not be internalized as efficiently as our protein.

The importance of removing dye contaminants from protein samples can be demonstrated by comparative internalization of protein and free-dye samples. Figure 3c shows cells electroporated with the original KF-Cy3b sample containing 18 % dye contamination, versus cells electroporated with the corresponding amount of free dye. Both the visual examples and the intensity histograms of segmented cells show that the two samples exhibit similar levels of fluorescence. These results suggest that the contaminating dye comprises a significant, and sometimes the major, proportion of fluorescence observed. In contrast, comparative internalization of dye-purified KF-Cy3b (containing 1 % dye contamination) versus the corresponding amount of free dye shows a significant difference in intensity distribution between the two samples. In particular, cells loaded with the amount of free dye corresponding to 1 % contamination exhibit intensities on the level of cellular autofluorescence (fluorescence of empty cells), and well below the level of KF-Cy3b signal. It can be concluded that, at least for this pair of protein and organic dye, 1 % dye contamination does not compromise cell loading with the labeled protein, and constitutes a workable condition under which one can be confident that the internalized fluorescence observed corresponds to the protein of interest and not the dye contaminant.

### Non-internalized fluorescence

**Fig. 4:**
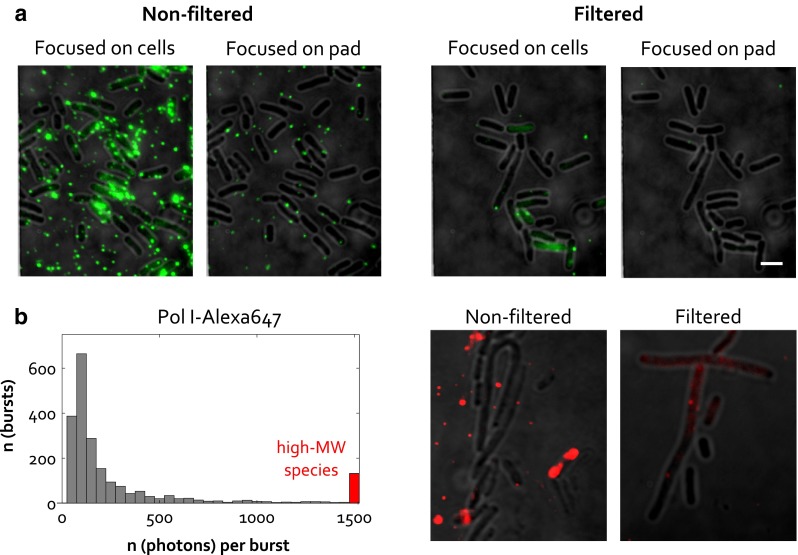
Use of cell filtration to remove non-internalized fluorescence for both monomeric (**a**) and aggregation-prone samples (**b**). **a** Example fields of view for internalization of 500 nM KF-Cy3b, without and with a cell-filtration step following cell recovery and washing. 3 steps of cell resuspension and centrifugation at 800×*g* were used, with 0.22 μm filter. Shown are fields of view in which fluorescence is focused either at the level of cells or at the level of the agarose pad, revealing non-internalized fluorescent contaminants that are removed by filtration. Electroporation at 1.0 kV voltage, near-TIRF mode, 532-nm excitation at 1 mW, 50 ms exposure. *Scale bar* 3 μm. **b**, *left* A histogram of the number of photons per burst for full-length Pol I-Alexa647 passing through the confocal volume, indicating the oligomeric state of the protein in solution. The Pol I-Alexa647 sample was electroporated in water under the same conditions as in the cell electroporation experiment. The *bar* containing high-molecular weight species (>1,500 photons per burst), very likely corresponding to Pol I aggregates, is denoted in *red*. **b**, *right* Internalization of Pol I-Alexa647, without (l.5 μM) and with the cell filtration step (150 nM). Removal of high-intensity fluorescence spots, corresponding to Pol I aggregates, can be observed. Electroporation at 1.8 kV voltage, widefield mode, 637-nm excitation at 100 μW (non-filtered) or 1 mW (filtered sample), 50 ms exposure

Although electroporation allows high internalization efficiencies to be achieved, the amount of non-internalized fluorescent molecules that remain in cell suspension after electroporation is typically much higher than the amount of internalized molecules. In the case of DNA fragments, virtually all of the non-internalized fluorescence can be removed by cell washing with simple buffers. In contrast, proteins may often bind non-specifically to the outer cell membrane, thus requiring additional components in the washing buffer to help remove non-internalized fluorescence. Such components include salt, to help break the electrostatic interactions between the protein molecules and the cell membrane, and non-ionic detergent, to counter hydrophobic interactions. However, we noticed that cell washing was not always sufficient to achieve a clean agarose pad background with electroporated cells, or a clean non-electroporated control (exhibiting intensities on the level of empty cells). More thorough removal of non-internalized fluorescence is sometimes necessary, and can be achieved by cell filtration using a 0.22 μm filter. Figure 4a shows the effect of filtration on the appearance of the level of non-internalized fluorescence. Notably, fluorescence contamination is reduced both at the level of the cells and at the level of the agarose pad, suggesting that both the molecules that bind non-specifically to the protein and the molecules that float in cell suspension are removed.

Whilst KF-Cy3b is a monomeric sample not prone to aggregation (data not shown), we demonstrate that cell filtration is also a convenient means of removing protein aggregates from cell suspension. As an example, full-length Pol I-Alexa647 is prone to aggregation in solution, and this tendency is increased under conditions of electroporation (Fig. 4b). This can be demonstrated by exposing Pol I-Alexa647 to electroporation conditions in the absence of cells, and analysing the sample in a confocal microscope set-up. The size of the fluorescence bursts that pass through the confocal volume is indicative of the oligomeric state of the protein (Hillger et al. 2007; Puchalla et al. 2008), and we observe a significant fraction of high-molecular weight species. Unsurprisingly, when Pol I-Alexa647 is used for internalization by electroporation, bright non-internalized spots are observed that correspond to protein aggregates. If a cell filtration step is added to the protocol, the non-internalized spots are removed and cell-internalized fluorescence is revealed, corresponding to the internalized monomeric fraction of Pol I.

### An example of optimized protein internalization

**Fig. 5:**
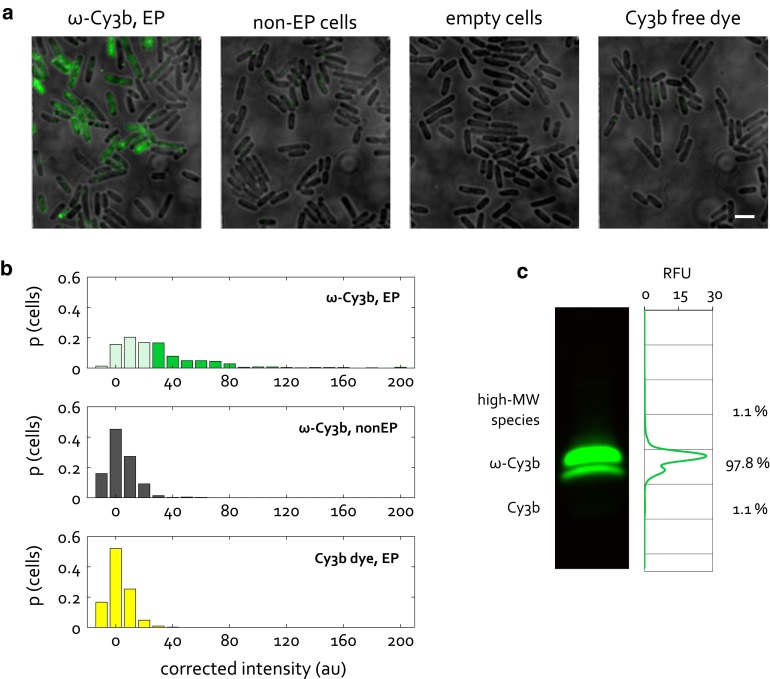
Internalization of RNAP ω subunit. **a** Example fields of view for internalization of 2.5 μM RNAP ω, at 1.4 kV voltage. Widefield mode, 532-nm excitation at 1 mW, 50 ms exposure. Non-EP and empty cells are defined as previously. Free dye was internalized at the same concentration as is present in the RNAP electroporated sample. *Scale bar* 3 μm. **b** Distribution of corrected cell-averaged intensities for samples in **a**, given in proportion of the total cell count. *Bars* corresponding to electroporated cells displaying intensities below the loading threshold are shown half-transparent. >400 cells per sample were segmented. **c** In-gel fluorescence of an SDS-PAGE gel of RNAP ω, showing ω-Cy3b as *two bands*, most likely due to an artefact of SDS-PAGE. The dye contamination band is not visible with the naked eye but is present at 1.1 % of total fluorescence

Using the above guidelines, we optimized the internalization of the 10-kDa omega (ω) subunit of bacterial RNA polymerase, labeled with Cy3b. Figure 5a, b shows that the protein gets internalized at high efficiency, whilst both the non-electroporated and empty-cell controls show virtually no fluorescence. In addition, contaminating dye is present at the level of 1 % (Fig. 5c), which when internalized in parallel with the ω sample, exhibits levels of fluorescence similar to the ones of empty cells. Hence, our ω-Cy3b sample was deemed to be a reliable sample to carry out further characterization of protein internalization by electroporation.

### Effect of voltage on internalization and viability

**Fig. 6:**
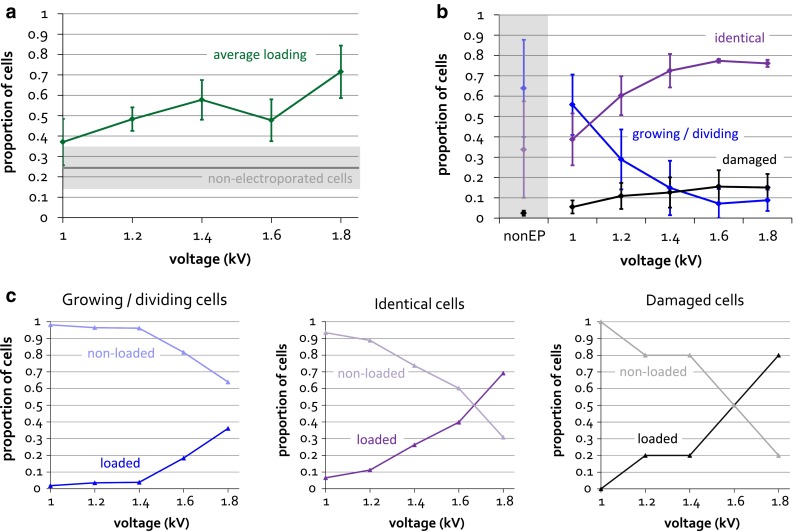
Effect of voltage on RNAP ω-Cy3b internalization efficiency (**a**) and cell viability (**b**). Cells were electroporated with 2.5 μM RNAP ω-Cy3b, at 1.00–1.80 kV, filtered, washed and recovered in EZ rich medium for 1–2 h. **a** Cells were electroporated, cell-averaged intensities measured and corrected for the mean intensity of empty cells. ‘Loaded’ cells correspond to cells exhibiting average fluorescence intensity higher than the intensity of non-electroporated cells plus 3 standard deviations. The proportion of loaded cells relative to the total cell count is plotted, with the standard deviation represented by *error bars*. Intensity of non-electroporated cells is shown for reference, with the standard deviation represented by the *grey box*. Imaging was done in near-TIRF mode, with 532-nm excitation at 600 μW, 100 ms exposure. >900 cells (3 independent internalizations of >300 cells each) were analysed for each voltage condition. **b** Cells were imaged in the white-light mode and classified as growing/dividing, identical or damaged cells. The non-electroporated cells are shown for reference, and the standard deviation is represented by *error bars*. >300 cells per voltage condition were analysed. **c** Correlation between cell loading and viability. Loading and viability were measured simultaneously for cells electroporated at different voltages, as in **a** and **b**. The effect of voltage on the proportion of loaded and non-loaded cells is shown for each class of cells separately: growing/dividing, identical and damaged cells. >300 cells per voltage condition were analysed

The electroporation voltage used is thought to affect the efficiency of electroporation, in terms of the number and the size of the membrane pores that are created. We wanted to see how the voltage used affects the efficiency of internalization of our optimized ω-Cy3b sample. We varied the electroporation voltage and measured the distributions of cell-averaged intensities resulting from protein internalization. We calculated the percentage of loaded cells by considering cells that are significantly (by at least 3 standard deviations) brighter than empty cells. As expected, increased voltage leads to increased loading (Fig. 6a), although there is significant variation in loading from experiment to experiment. Notably, the non-electroporated control occasionally includes cells that exhibit high fluorescence, most likely corresponding to damaged cells whose membranes are compromised and can allow internalization even without electroporation. This phenomenon increases the effective background level of fluorescence, which has to be taken into account when interpreting the loading results. Hence, while ‘absolute’ loading varies from 40 to 70 %, loading corrected for non-electroporated cells is in the range from 15 to 45 %.

In addition, we explored the effect of voltage on cell viability. We examined cells in the white-light mode over 1–2 h following electroporation and recovery, and observed four different classes of cells: (1) dividing cells, which divided into daughter cells during the course of imaging; (2) growing cells, which grew in length but did not divide into daughter cells; (3) identical cells, which neither divided nor grew, but their membranes appeared intact; and (4) damaged cells, with visibly damaged cell membranes. We quantified the proportion of cells in each class, and observed that the number of growing and dividing cells decreased with voltage, whilst the number of identical and damaged cells increased (Fig. 6b). To rule out the possibility that it is only damaged cells that get loaded with fluorescent molecules, we set out to determine the level of correlation between cell viability and internalization, by performing the viability and loading measurements simultaneously. As expected, our results show that the applied voltage positively affects loading for all classes of cells (Fig. 6c). The effect of voltage on loading is stronger for damaged cells than for identical cells, and stronger for identical cells than for growing and dividing cells, suggesting that there is some level of correlation between cell internalization and viability. However, since the percentage of damaged cells is the sample is low (2 % at 1.4 kV voltage), the majority of loaded cells under any voltage condition will correspond to identical, growing and dividing cells.

## Discussion

In this paper, we present an optimized protocol for using electroporation to internalize organically labeled proteins in living bacteria. We explored and defined optimal buffer conditions compatible with efficient loading of cells with the labeled protein of interest. Specific buffering components and salts can be varied, as long as the overall ionic strength of the buffer remains low. When higher ionic strengths are required to ensure stability and activity of the protein of interest, we suggest storing the protein in its optimal buffer at high concentration, which allows a larger dilution factor when the protein sample is added to cell suspension for electroporation. Alternatively, the protein sample can be diluted into a low-ionic strength buffer and concentrated just prior to electroporation. Notably, the required level of internalization (e.g. for single-cell ensemble fluorescence studies versus single-molecule fluorescence studies) will determine the amount of sample that has to be used, and hence the maximum ionic strength that can be present in the buffer. Similarly, higher ionic strengths can be used at lower electroporation voltages (1.00–1.20 kV), and glycerol can be added to slightly increase the threshold of suitable ionic strength. Hence, the optimal buffer conditions will have to be adapted based on both the characteristics of the protein of interest and the desired application.

We also present a protocol that allows removal of dye contamination from protein samples prior to electroporation. The contaminating dye can be present in a sample either as a ‘free’ unreacted dye, or bound non-specifically to the protein. His-tag purification of dye contaminants works against both types of contamination, although it is likely more effective in removing the free dye. From a theoretical standpoint, the free dye contamination is much more problematic in terms of internalization by electroporation, as free dye gets internalized in preference to the protein. The non-specifically bound dye is not always problematic; it is expected to be internalized at an efficiency similar to the internalization efficiency of the protein, and may or may not dissociate from the protein once internalized. Our experiments show that a *free* dye contamination of 1 %, which corresponds to the worst scenario given the 1 % *total* dye contamination quantified from SDS-PAGE, does not significantly affect the cell-averaged fluorescence observed in cells electroporated with a fluorescently labeled protein. Since the highest acceptable level of dye contamination will depend on the specific protein and organic dye that are used, appropriate controls of side-by-side internalization of protein and free dye should be carried out for each new labeled protein under study.

Following electroporation, non-internalized fluorescent molecules need to be removed from the cell suspension. We showed that filtration constitutes a more effective means of removing non-internalized fluorescence than just a cycle of centrifugation and cell resuspension, presumably due to the constant flow of buffer that exerts a force on the membrane-bound molecules. Knowledge of the properties of the protein of interest will facilitate the choice of washing procedures. For example, salts or non-ionic detergents may help break electrostatic or hydrophobic interactions, respectively, and treatment with a low concentration of protease (such as Proteinase K) can be used to degrade membrane-bound fluorescent molecules that cannot be removed by other means. In our experiments, we also observed cells with compromised membranes that take up fluorescent molecules in the absence of electroporation. As internalized molecules cannot be removed by cell washing or filtration, these cells contribute to the background level of fluorescence; however, they do not compromise our analysis of protein internalization. In terms of unstable protein samples that are prone to aggregation, we show that cell filtration can be highly effective for removing non-internalized protein aggregates from cell suspension. Importantly, due to their large size most of the aggregates will not be internalized, and most of the internalized fluorescence corresponds to monomeric species, as confirmed by single-molecule photobleaching experiments (data not shown).

Previously, we showed that a significant fraction of the internalized proteins retain their activity in vivo, and can perform their intended functions in the cytoplasm (Crawford et al. 2013). We also noticed a general effect of the applied voltage on the internalization efficiency, and we could observe and quantify the even distribution of the total fluorescence between daughter cells upon cell division. Here, we were able to describe in detail the linear correlation between internalization efficiency and the applied voltage, consistent with the idea that higher voltage is associated with an increase in the number and size of pores in the bacterial membrane. However, considerable variation in internalization efficiency is observed between experiments, with some non-electroporated cells exhibiting a significant amount of fluorescence. It is likely that these cells feature membranes that have been overly compromised during the induction of electrocompetency. As expected, viability decreases with the applied voltage, as the greater number and size of membrane pores is likely to be more detrimental to cell integrity. We assume growing and dividing cells to be healthy, and identical cells to be in a viable but stressed state, resulting from the electroporation shock and in need of further recovery to resume growth and division. However, a more robust viability assay would be needed to confirm these assumptions, such as testing for expression of a fluorescently labeled protein in real time. Cells exposed to filtration display lower viability than cells washed only by cycles of centrifugation and resuspension (Crawford et al. 2013), but this may be a necessary price to pay for the removal of non-internalized fluorescence. We show that the major fraction of loaded cells always corresponds to non-damaged cells, although most of the damaged cells will appear loaded at higher voltages. However, as only a small proportion of cells are visibly damaged, most of the loaded cells would be suitable for physiologically relevant studies. If absolute viability is required, the loaded cells of interest can always be tested for their viability by following their division after recoding the fluorescence data.

Our electroporation-based method provides a means of internalizing any fluorescently labeled protein of significant size (up to 60 kDa) into live bacteria. Although the optimal conditions for internalization will have to be adapted for each protein of interest, and appropriate tests will need to be performed to ensure that the specific protein retains its activity in vivo, our study provides useful guidelines on how to achieve this. Depending on the copy number and the nature of the protein of interest, internalized labeled proteins can either compete with the endogenous pool of proteins or fully replace it (if the encoding gene can be deleted). For any experiment, it is important to determine the ratio of the internalized versus endogenous copy numbers of the protein of interest, and electroporation provides a tool to control this ratio. Successful internalization of organically labeled proteins will then fuel a plethora of new studies. For example, due to the high photostability of organic fluorophores, the dynamics and localization of the protein of interest can be studied on a much longer timescale than previously possible (up to several minutes). Moreover, internalization of doubly labeled proteins allows single-cell and single-molecule FRET studies in live cells (Crawford et al. 2013). In vivo FRET studies have the potential to probe the physiological structure of proteins that have been studied using conventional structural biology techniques, and it will be possible to compare in vivo generated distances with the ones expected from the in vitro structures. In the long term, along with the discovery of improved organic dyes and the advancements in fluorescence imaging, real-time investigation of conformational dynamics of molecular machines in a variety of cells should become a reality.
